# Supplementary material for: In vivo guiding nitrogen-doped carbon nanozyme for tumor catalytic therapy
Source: Nat Commun. 2018 Apr 12;9:1440. doi: 10.1038/s41467-018-03903-8 (PMC5897348; doi:10.1038/s41467-018-03903-8)
Supplement: Supplementary file 1 — Supplementary Information [file 41467_2018_3903_MOESM1_ESM.pdf]

Supplementary Information

In vivo Guiding Nitrogen-doped Carbon Nanozyme for Tumor Catalytic Therapy

Fan *et al.*

Supplementary Figures

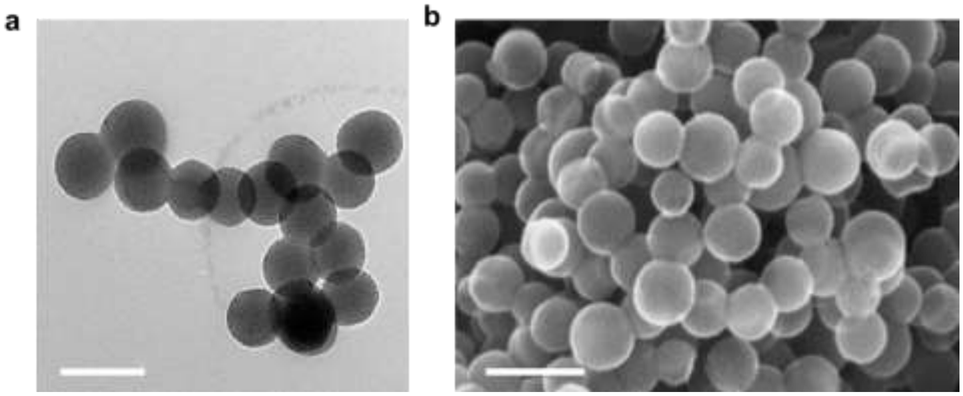

Supplementary Figure 1. (a) TEM and (b) SEM images of N-PCNSs-3. Scale bar: 200 nm.

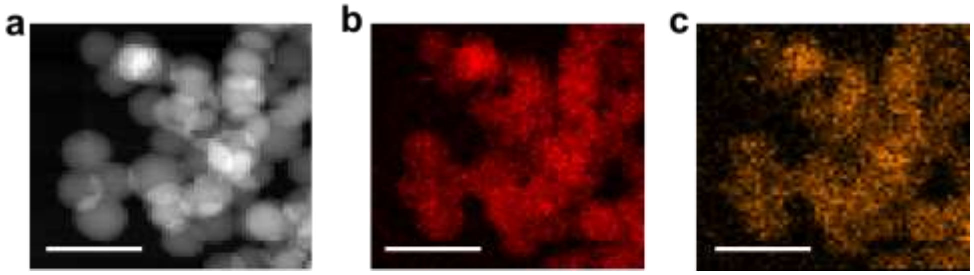

Supplementary Figure 2. Elemental mappings of N-PCNSs. Mapping area (**a**) and the results for C K-edge (**b**) and N K-edge (**c**) signals of N-PCNSs-3. Scale bar: 200 nm.

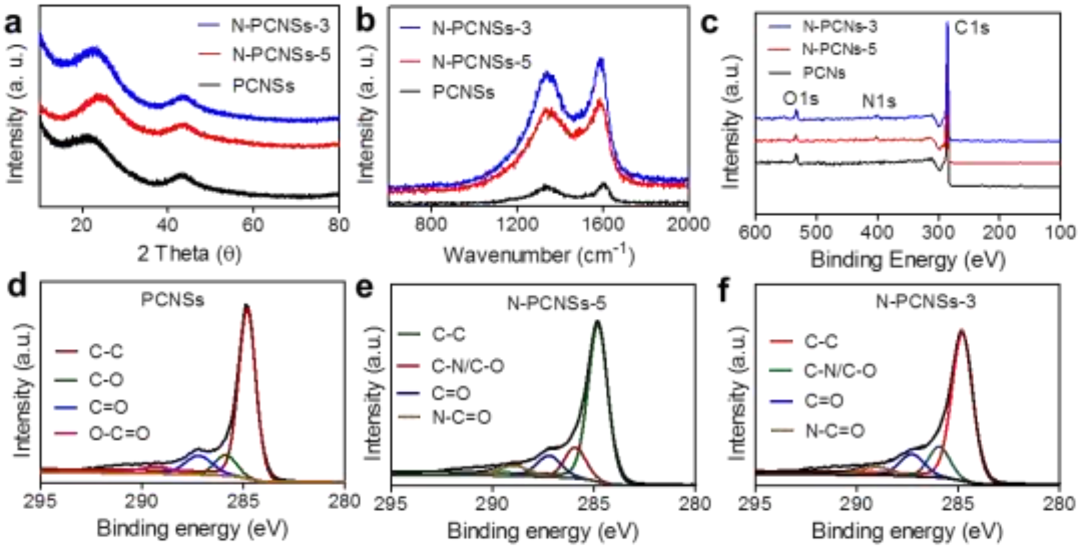

Supplementary Figure 3. Characterizations of N-PCNSs. a, XRD spectra of N-PCNSs. **b**, Raman spectra of N-PCNSs. **c**, XPS spectra of N-PCNSs. **d**, **e** and **f** are C1s spectra for the PCNSs, N-PCNSs-5 and N-PCNSs-3, respectively.

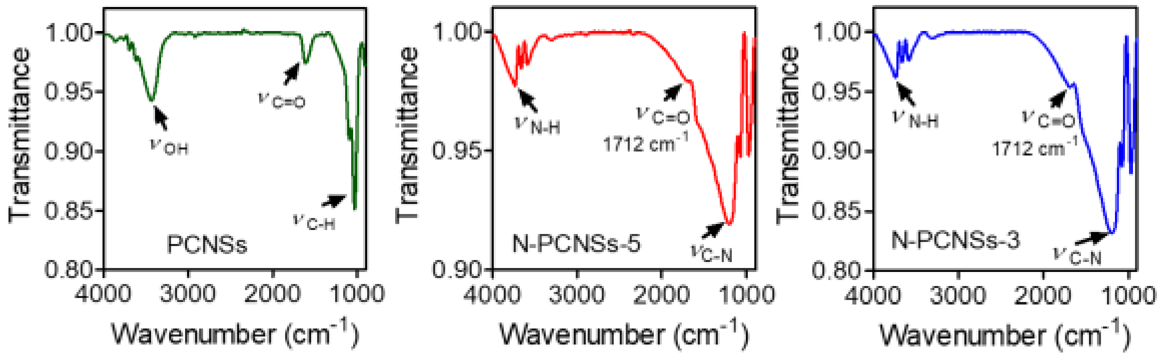

Supplementary Figure 4. FTIR spectra of PCNSs, N-PCNSs-5 and N-PCNSs-3.

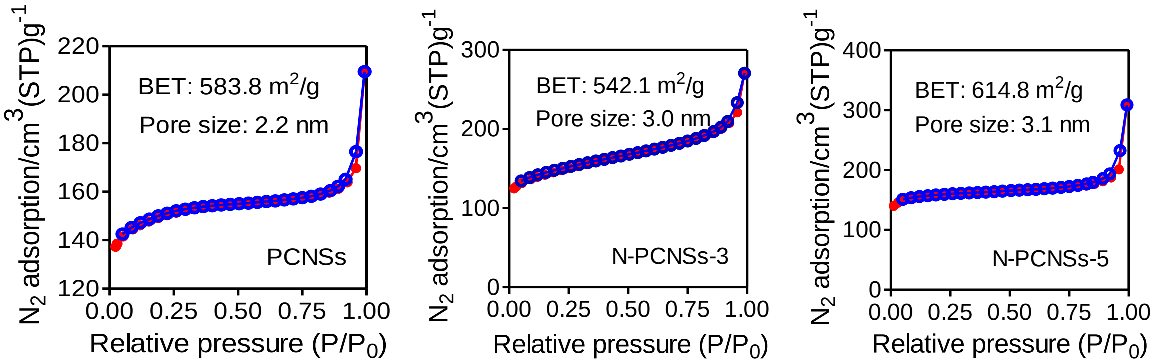

Supplementary Figure 5. Nitrogen adsorption-desorption isotherms of PCNSs, N-PCNSs-3 and N-PCNSs-5, and corresponding BET surface area and pore-size data.

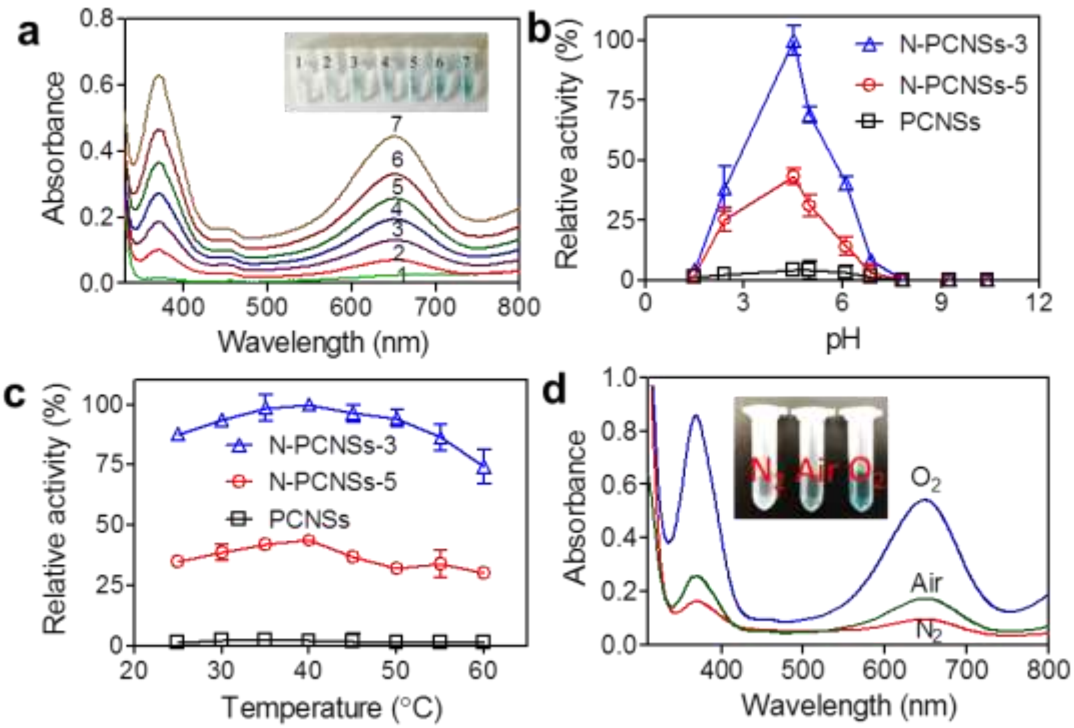

Supplementary Figure 6. Characterization for OXD-like activity. a, The absorbance spectra and visual color changes of TMB (0.416 mM) in the presence of different concentrations of N-PCNSs-3 after 30 min incubation (0.1 M NaAc buffer, pH 4.5): (1) 0 μg mL^{-1}, (2) 12.5 μg mL^{-1}, (3) 25 μg mL^{-1}, (4) 32.5 μg mL^{-1}, (5) 50 μg mL^{-1}, (6) 75 μg mL^{-1}, (7) 100 μg mL^{-1}. **b** and **c**, The OXD-like activities of PCNSs, N-PCNSs-5 and N-PCNSs-3 are dependent on both pH and temperature. **d**, Direct oxidation of TMB by N-PCNSs-3 in N_2, air and O_2 atmosphere. Error bars shown represent the standard error derived from three independent measurements.

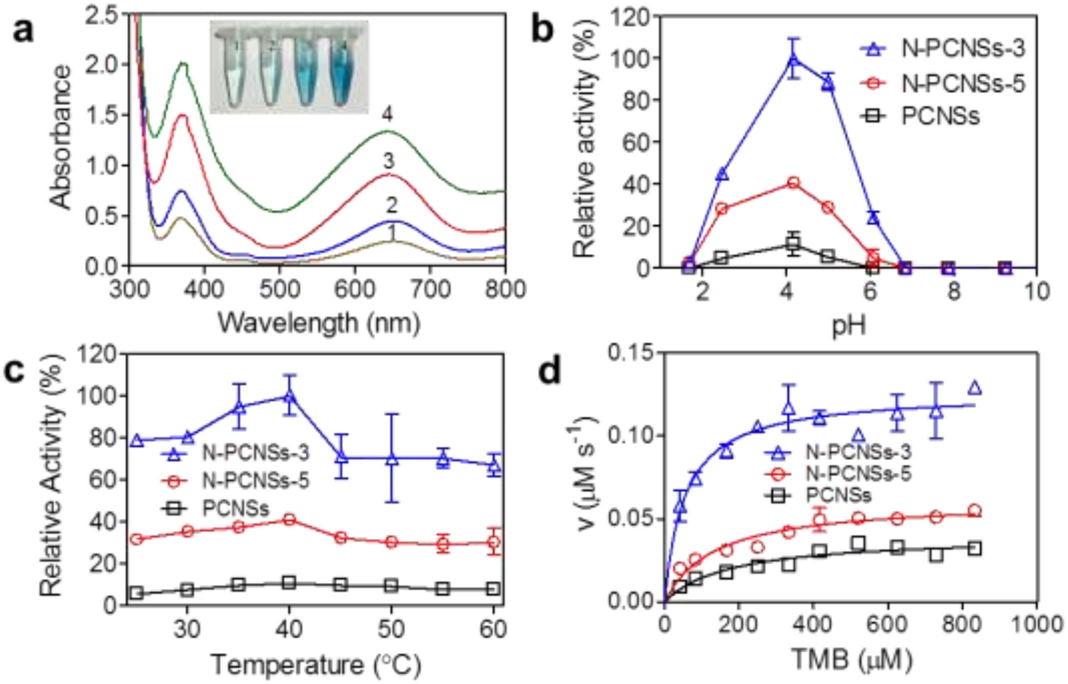

Supplementary Figure 7. Characterization for POD-like activity. a, The absorbance spectra and visual color changes of TMB: (1) TMB + H_2O_2, (2) TMB + H_2O_2 + PCNSs and (3) TMB+ H_2O_2 + N-PCNSs-5 (4) TMB+ H_2O_2 + N-PCNSs-3 in 0.1 M NaAc buffer pH 4.5 at 40 ^{o}C after 10 min incubation. TMB: 0.416 mM, H_2O_2: 0.5292 M, PCNSs, N-PCNSs-5 and N-PCNSs-3: 25 μg mL^{-1}. **b** and **c**, The POD-like activity of the PCNSs, N-PCNSs-5 and N-PCNSs-3 is dependent on pH and temperature. **d**, Kinetics for PCNSs to TMB substrate. Error bars represent the standard error derived from three independent measurements.

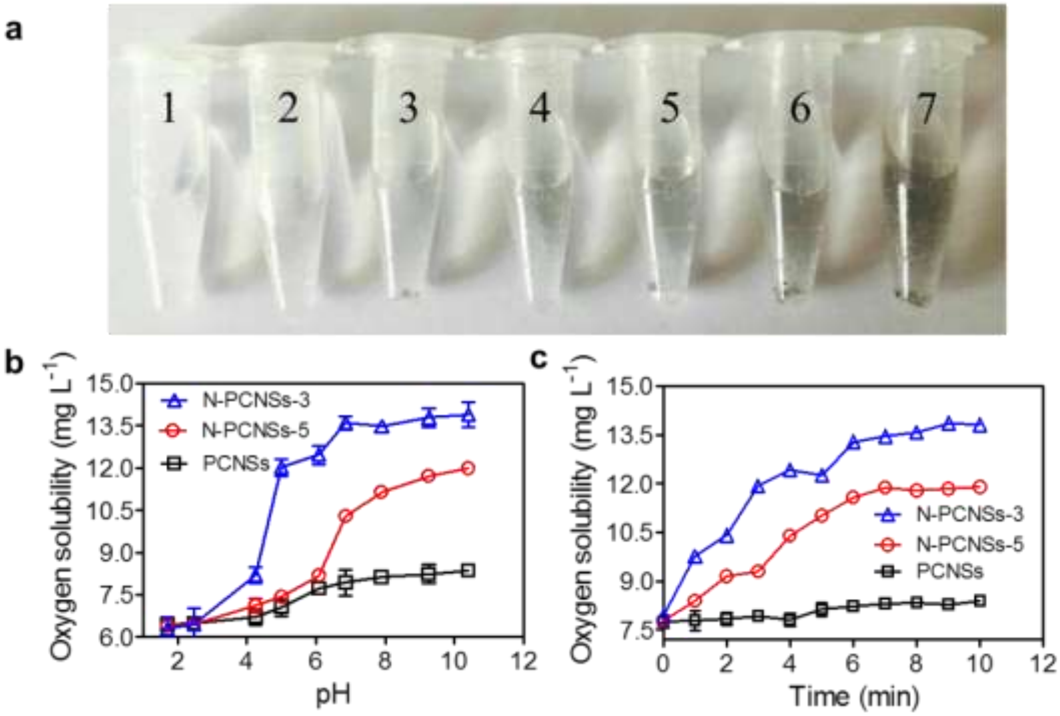

Supplementary Figure 8. Characterization for CAT-like activity. a, Images of systems reacting for 10 min at pH 7.0 in the presence of different concentrations of N-PCNSs-3. (1) 0 µg mL^{-1}, (2) 12.5 µg mL^{-1}, (3) 25 µg mL^{-1}, (4) 32.5 µg mL^{-1}, (5) 50 µg mL^{-1}, (6) 75 µg mL^{-1}, (7) 100 µg mL^{-1} with 0.882 M H_2O_2. **b**, Oxygen generation catalysis by PCNSs, N-PCNSs-5 and N-PCNSs-3 in different pH buffer solutions after 10 min incubation. Reaction conditions: 25 µg mL^{-1} PCNSs, N-PCNs-5 or N-PCNSs-3; and 0.882 M H_2O_2. **c**, Effect of time on the oxygen generation from H_2O_2 decomposition. Reaction conditions: 25 µg mL^{-1} PCNSs, N-PCNSs-5 or N-PCNSs-3; and 0.882M H_2O_2. Error bars shown represent the standard error derived from three independent measurements.

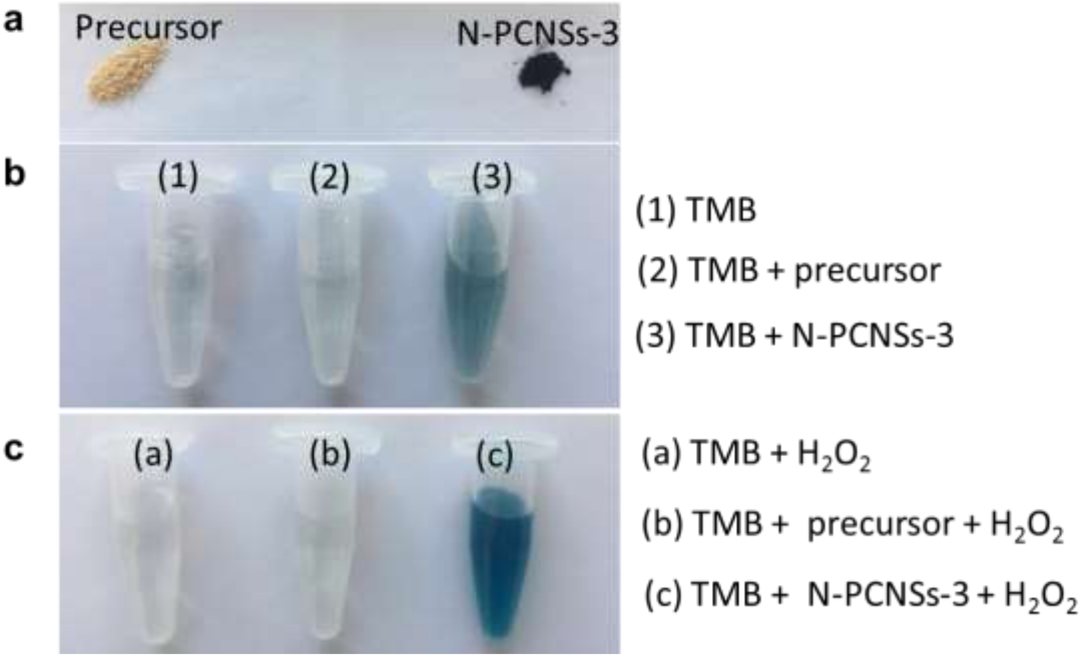

Supplementary Figure 9. Comparison of Activity of N-PCNSs and their precursor. a, The photographs of the precursor and N-PCNSs. **b**, The OXD-like activity in the reaction containing 0.416 mM TMB and 25 µg mL^{-1} precursor or N-PCNSs. **c**, The POD-like activity of in the reaction containing 0.416 mM TMB, 0.5292M H_2O_2 and 25 µg mL^{-1} precursor or N-PCNSs.

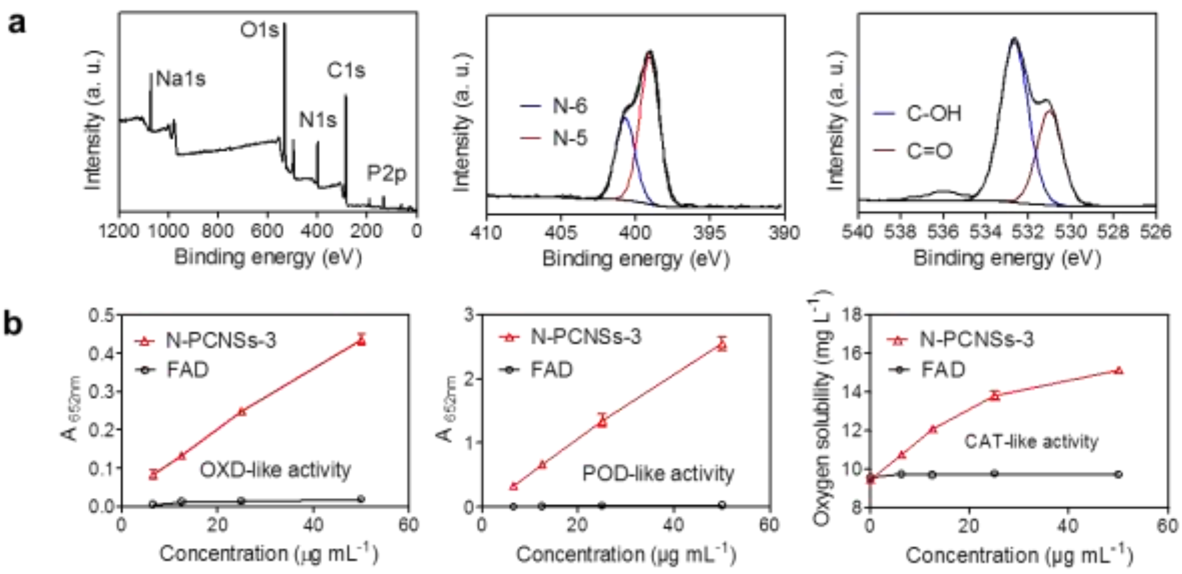

Supplementary Figure 10. Characterization and enzyme-like activity of FAD. **a**, XPS spectra, N1s and O1s deconvolution peak of FAD. **b**, The enzyme-like activities of FAD. All materials were used at 25 μg mL^{-1} with 0.882 mM H_2O_2 and 0.416 mM TMB. Error bars shown represent the standard error derived from three independent measurements.

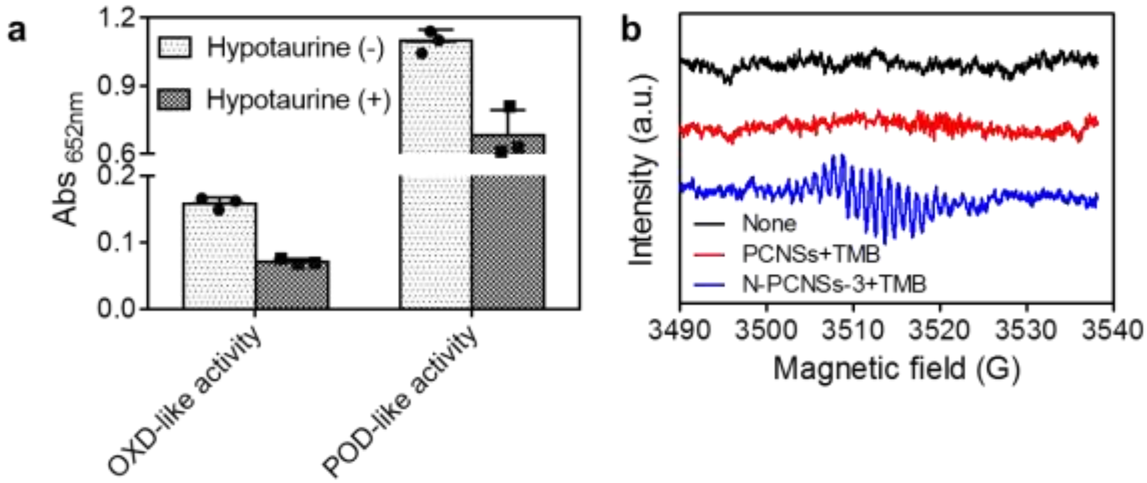

Supplementary Figure 11. Free radical quenching and identification in OXD-like and POD-like activities. **a**, The effect of hypotaurine on the enzyme-like reaction of 25 μg mL^{-1} N-PCNSs-3, 0.416 mM TMB, 0.1764 mM H_2O_2 with or without 4% (w/w) hypotaurine. The reaction time was 20 min for OXD-like activity and 10 min for POD-like activity. Mean values and error bars are defined as mean and *s.d.*, n = 3. **b**, ESR characterization shows that N-PCNSs directly reacts with TMB via OXD-like activity.

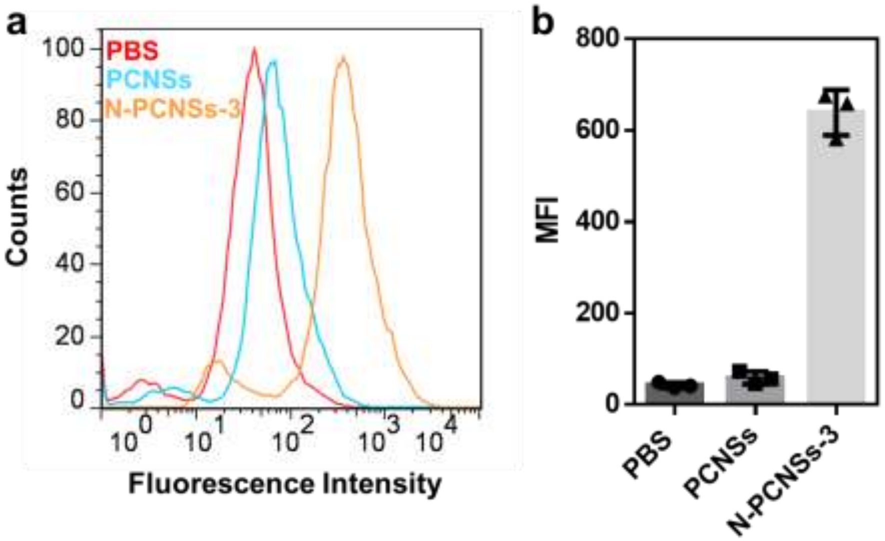

Supplementary Figure 12. Quantification of cellular ROS with flow cytometry. a, Spectrum for fluorescence counting by flow cytometry. **b**, Quantification analysis for the results from **a**. Mean values and error bars are defined as mean and *s.d.*, n = 3.

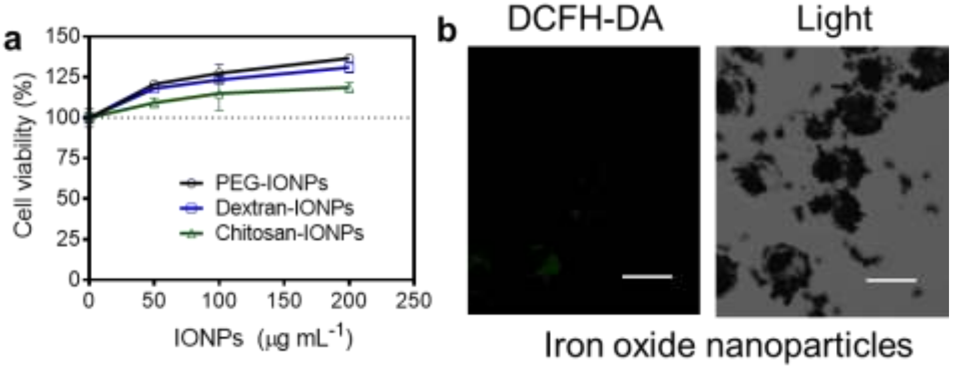

Supplementary Figure 13. Effects of iron oxide nanoparticles (IONPs) on cell viability and cellular ROS. a, IONPs with different modifications exhibited no toxicity to HepG2 Cells. **b**, IONPs did not significantly increase the ROS levels in HepG2 cells. Scale bar: 10 µm. Mean values and error bars are defined as mean and *s.d.*, n = 6.

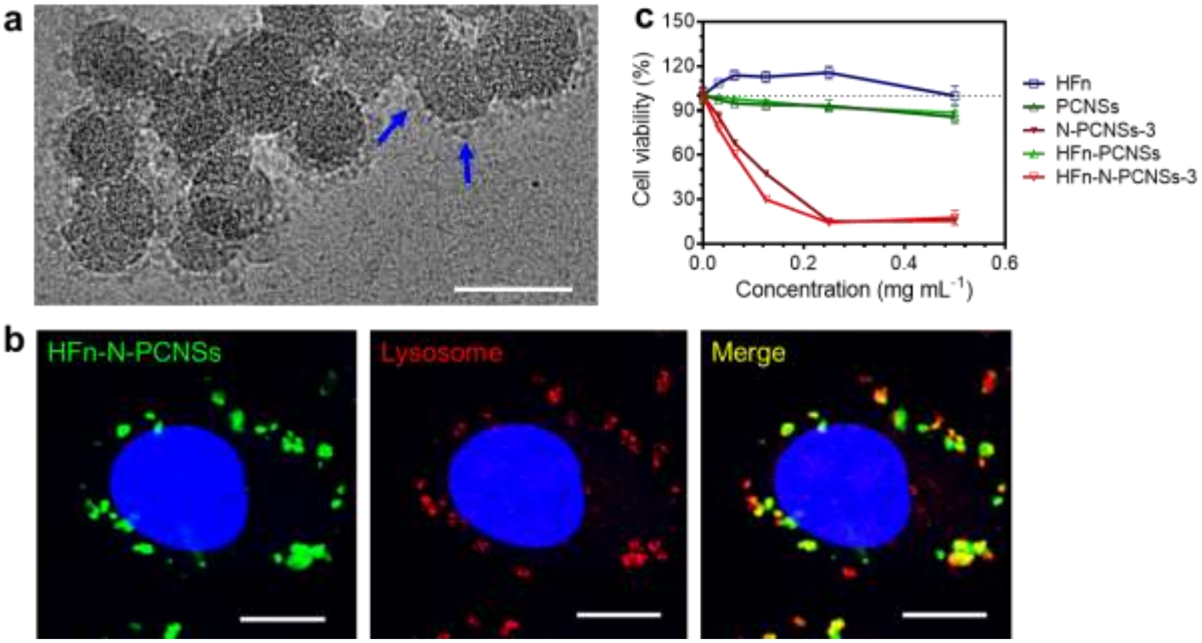

Supplementary Figure 14. HFn conjugation, internalization and effect on cell viability. a, HFn modification of N-PCNSs characterized with cryo-TEM. Scale bar: 100 nm. **b**, HFn facilitates lysosomal localization of nanozymes. Lysosomes are visualized using LysoTracker. The Pearson's coefficient of N-PCNSs and lysosome was determined as 0.8912. Scale bar: 10 μm. **c**, Following modification, N-PCNs retain their ability to reduce HepG2 cell viability. Mean values and error bars are defined as mean and *s.d.*, n = 6.

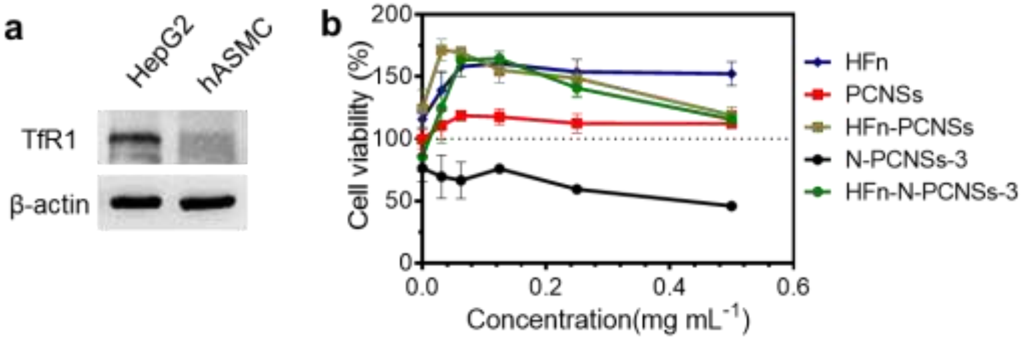

Supplementary Figure 15. Effect of HFn-N-PCNSs on cells without TfR1. **a**, Human aortic smooth muscle cells (hASMCs) without TfR1 expression. **b**, The cell toxicity effect of HFn-N3 was absent on hASMCs. Mean values and error bars are defined as mean and *s.d.*, n = 6.

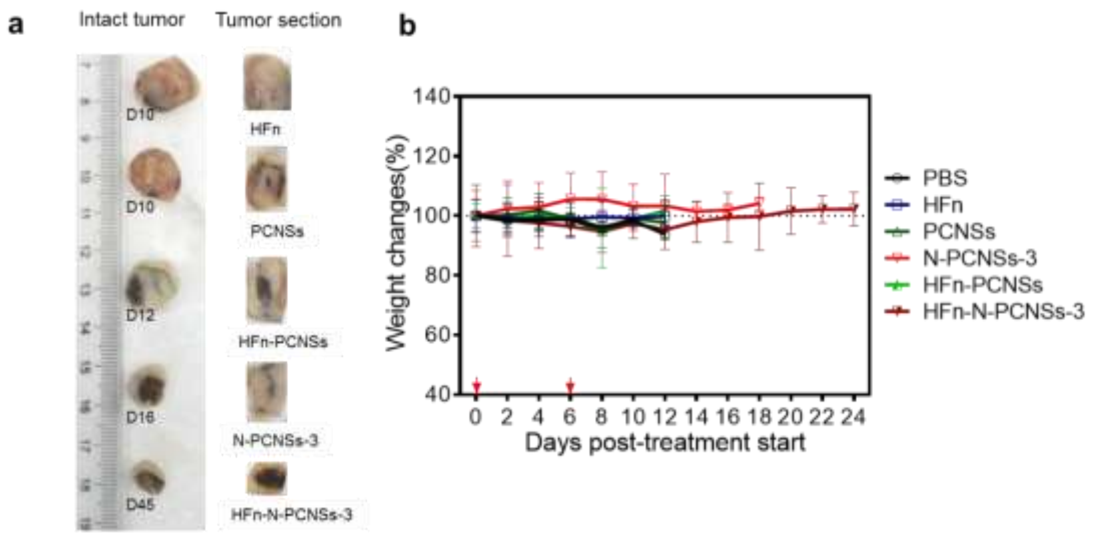

Supplementary Figure 16. Anti-tumor activity and biosafety evaluation of N-PCNSs using HFn coordination. Tumor size (**a**) and body weight changes (**b**) in N-PCNSs treated HepG2 tumor bearing mice via *i.t.* injection. Mean values and error bars are defined as mean and *s.d.*, n = 5.

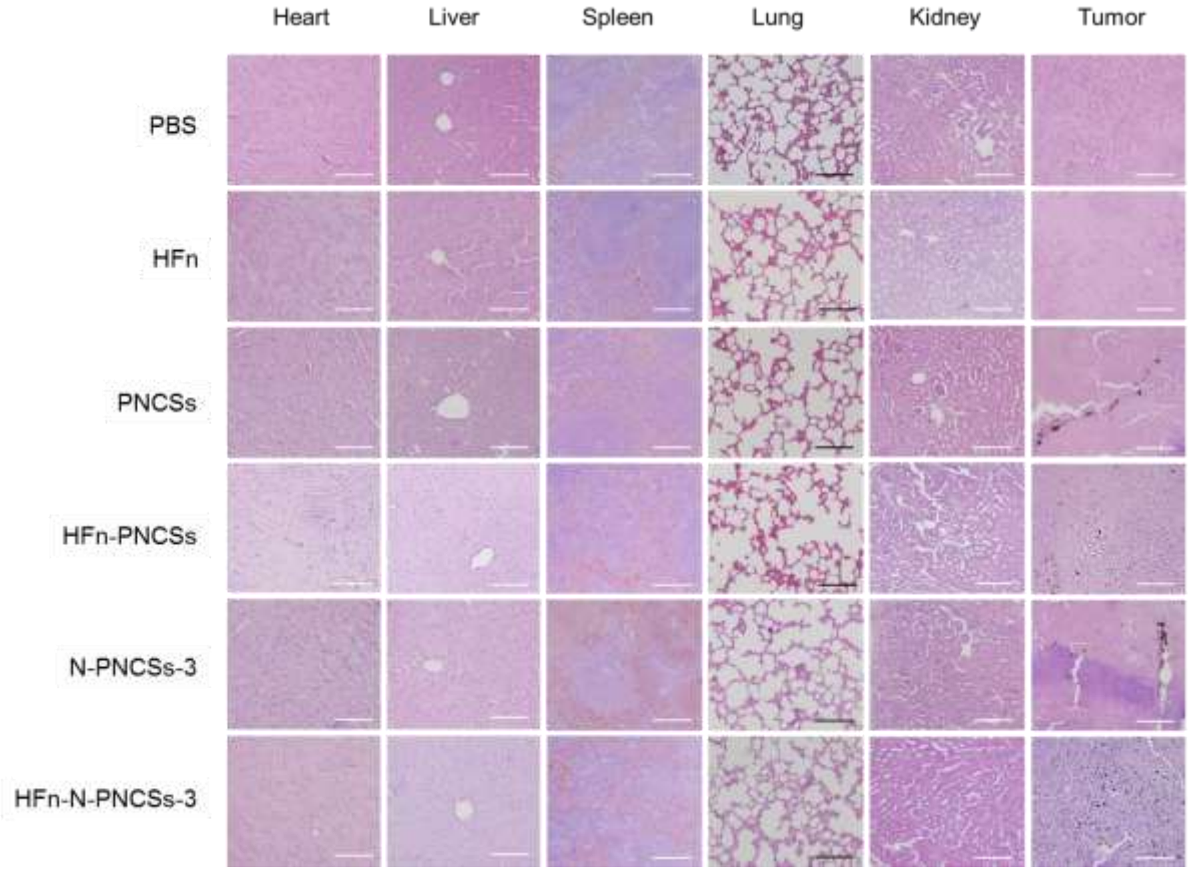

Supplementary Figure 17. H&E staining analysis of the main organs and tumors of the N-PCNSs treated mice. Scale bar is 200 μm for heart tissues, and 300 μm for other organ tissues.

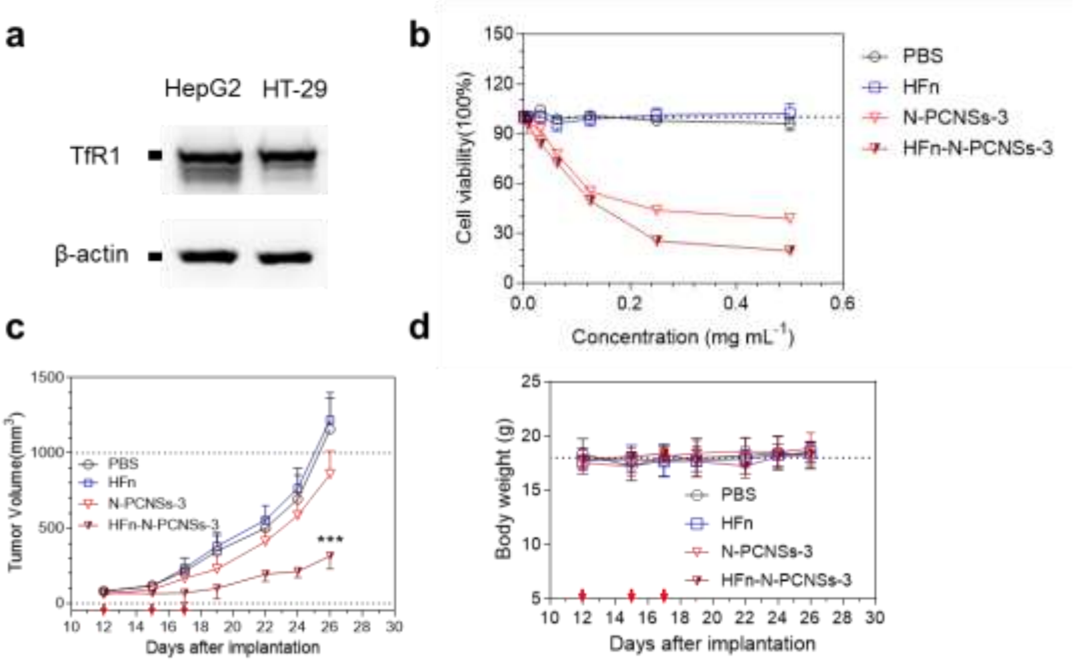

Supplementary Figure 18. **HFn-N-PCNS-based HT-29 tumor therapy. a**, Western blot analysis of TfR1 expression in HT-29 and HepG2 tumor cells. **b**, Effect of N-PCNSs on HT-29 cell viability. **c**. Tumor volume after *i.v.* treatment with HFn-N-PCNSs. Statistical significance is assessed by unpaired Student's *t* test compared to the control groups. ***p < 0.001. **d**. Body weight changes of the treated mice following *i.v.* injection of HFn-N-PCNSs. Mean values and error bars are defined as mean and *s.d.*, n = 5.

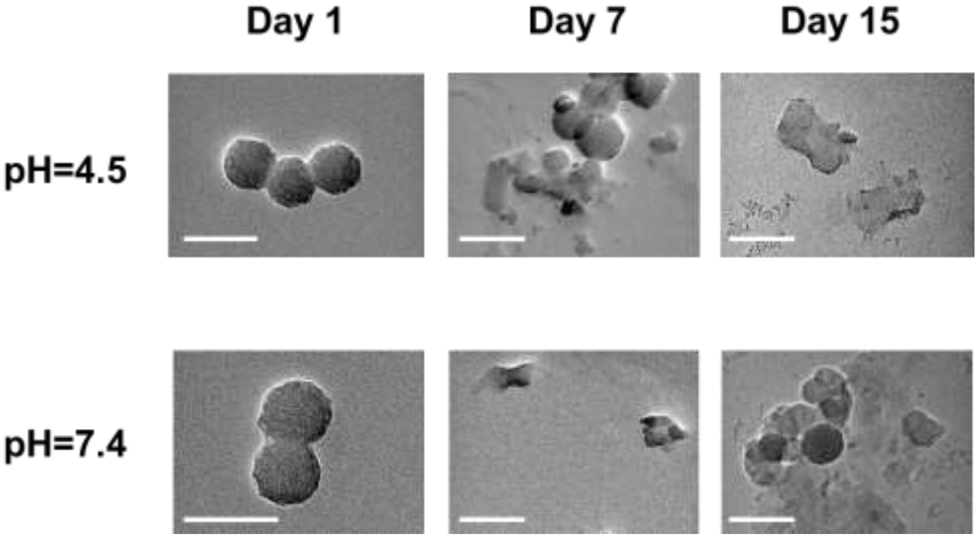

Supplementary Figure 19. Biodegradation of N-PCNSs. TEM images of N-PCNSs after biodegradation in neutral (pH = 7.4) and acidic (pH = 4.5) buffers for 1, 7, and 15 days. Scale bar: 100 nm.

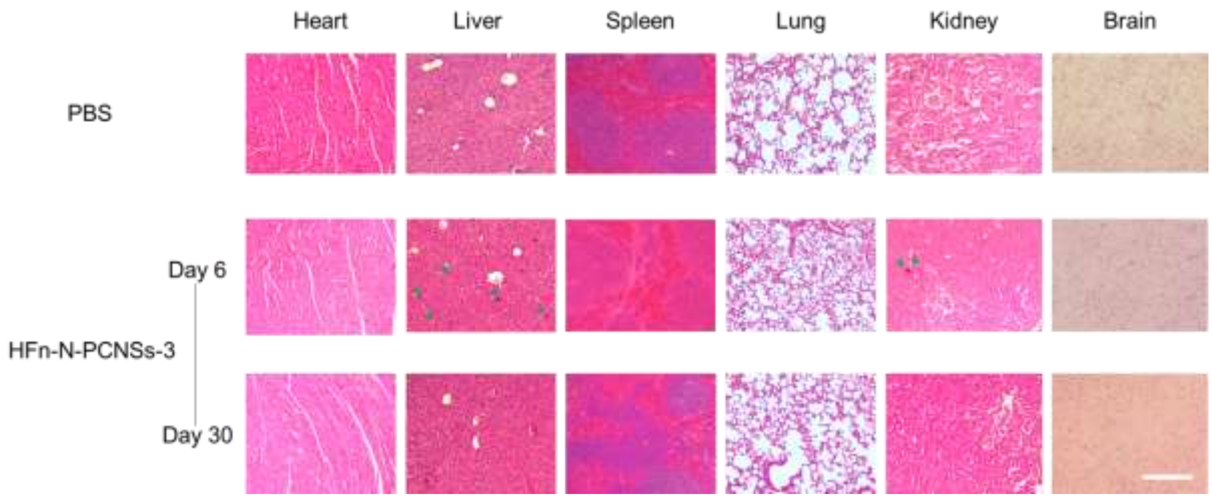

Supplementary Figure 20. Pathological analysis of the potential toxicity influence of HFn-N-PCNSs on healthy mouse organs. The analysis of mice treated with HFn-N-PCNSs was performed on days 6 and 30 following carbon materials *i.v.* injection. The green arrows indicate the position of the HFn-N-PCNSs nanoparticles. Scale bar: 300 μm.

Supplementary Tables

Supplementary Table 1. Carbon, oxygen and nitrogen contents [atom%] in the studied samples obtained from X-ray photoelectron spectroscopy (XPS) and I_D/I_G ratio form Raman spectra.

Catalyst	C atom%	N atom%	O atom%	I_D/I_G
**PCNSs**	93.66	0	6.34	0.934
**N-PCNSs-5**	93.09	2.85	4.06	0.925
**N-PCNSs-3**	92.04	3.37	4.58	0.926

Supplementary Table 2. Relative surface concentrations of nitrogen species obtained by fitting the N1s core-level XPS spectra.

Catalyst	N atom%	N-5 atom %	N-6 atom%	N-Q atom%	N6/N5
**N-PCNSs-5**	2.85	0.33	0.65	1.87	1.98
**N-PCNSs-3**	3.37	0.33	0.85	2.19	2.58

Supplementary Table 3. The Michaelis-Menten constant (K_M) and maximum reaction rate (V_{max}) of as prepared N-PCNSs with TMB as the substrate for OXD-like catalysis.

Catalyst	[E] (mg mL^{-1})	Substrate	K_M (mM)	V_{max} (10^{-8} M s^{-1})
**PCNSs**	2.5×10^{-2}	TMB	ND	ND
**N-PCNSs-5**	2.5×10^{-2}	TMB	0.095	0.27
**N-PCNSs-3**	2.5×10^{-2}	TMB	0.084	0.42

ND: not detected

Supplementary Table 4. The Michaelis–Menten constant (K_M) and maximum reaction rate (V_{max}) of as-prepared N-PCNSs with TMB and H_2O_2 as the substrates for POD-like catalysis.

Catalyst	[E] (mg mL^{-1})	Substrate	K_M (mM)	V_{max} (10^{-8} M s^{-1})
**PCNSs**	2.5×10^{-2}	TMB	2.254	4.47
**PCNSs**	2.5×10^{-2}	H_2O_2	471	5.52
**N-PCNSs-5**	2.5×10^{-2}	TMB	0.135	6.13
**N-PCNSs-5**	2.5×10^{-2}	H_2O_2	161	11.7
**N-PCNSs-3**	2.5×10^{-2}	TMB	0.0549	12.6
**N-PCNSs-3**	2.5×10^{-2}	H_2O_2	130	32.5

Supplementary Table 5. The Michaelis–Menten constant (K_M) and maximum reaction rate (V_{max}) of as-prepared N-PCNs with H_2O_2 as the substrate for CAT-like catalysis.

Catalyst	[E] (mg mL^{-1})	Substrate	K_M (M)	V_{max} (mg L^{-1} min^{-1})
**PCNSs**	2.5×10^{-2}	H_2O_2	0.6789	0.2525
**N-PCNSs-5**	2.5×10^{-2}	H_2O_2	0.1540	0.4720
**N-PCNSs-3**	2.5×10^{-2}	H_2O_2	0.06625	0.5143

Supplementary Discussion

As shown in Supplementary Fig. 3a, X-ray diffraction patterns displayed two peaks near 24° and 42°, presenting the characteristics for the (002) and (100) graphitic planes. The formation of the graphitic structure was further identified using Raman spectroscopy (Supplementary Fig. 3b). The results showed an intense and narrow band at 1590 cm^{-1} (G-band, the first-order scattering of the E2g vibrations observed for sp^2 domains in an ideal graphitic layer) and 1340 cm^{-1} (D-band, correlated to structural defects and partially disordered structure of sp^2 domains). The peak intensity ratios of D and G bands (I_D/I_G) for PCNSs, N-PCNSs-5 and N-PCNSs-3 were measured as 0.934, 0.925 and 0.926, respectively. These data indicated that the obtained carbon materials have been highly graphitized. XPS results showed that N-PCNSs-5 and N-PCNSs-3 were N-doped to 2.85 atom% and 3.37 atom%, respectively, while the PCNSs showed no nitrogen content (Supplementary Fig. 3c and Supplementary Table 1).

The bonding configurations of the nitrogen atoms of N-PCNSs were characterized by high resolution N 1s spectra. There were four peaks at 398.38 eV, 399.78 eV, 401.18 eV, and 402.88 eV, presenting pyridinic nitrogen (N-6), pyrrolic nitrogen (N-5), quaternary nitrogen (N-Q) and pyridine oxide or the oxidized nitrogen ($N\text{-}O_X$), respectively. Compared to N-PCNSs-3, the N-6, N-Q and $N\text{-}O_X$ configurations except N-5 in N-PCNSs-5 were decreased (Supplementary Table 2). This indicated that prolonging carbonization time at high temperature affected the amount and type of N-doping in carbon materials. The high-resolution O 1s spectrum of the N-PCNSs-3 revealed that the O atoms mainly were in three types, C=O (530.98 eV), C-OH (532.58 eV) and COOH (534.28 eV) (Fig. 1e), implying that oxygen-containing functional groups were present on the surface of the material.

The C 1s high resolution XPS spectrum of PCNSs (Supplementary Fig. 3d) exhibited four main peaks at 284.6, 285.1, 286.3, and 288.0 eV, which were assigned to C-C, C-O, C=O and O-C=O, respectively. In N-PCNSs-5 and N-PCNSs-3, the intensity of the C-O/C-N peaks was increased (Supplementary Figs. 3e and 3f), further indicating that N was doped into the carbon framework.

The FTIR spectrum of PCNSs showed that plenty of O functional groups, such as O-H and C=O, presented on the surface of PCNSs, indicating that hydroxyl or carboxyl groups were formed on the surface of PCNSs (Supplementary Fig.4). With the N-doping, we found that the characteristic absorption bands of N-H were stretching vibrations at 3770 cm^{-1}, as well as aromatic C-N stretching vibration at 1234 cm^{-1}. Moreover, the C=O bands located at 1712 cm^{-1} were also observed in N-PCNSs, and the band intensity was decreased with the prolong carbonation time in N-PCNSs-5 compared to N-PCNSs-3.

As shown in Supplementary Fig. 5, BET Isotherm was performed to get further insight about surface area and porous structure. The surface areas of PCNSs, N-PCNSs-3 and N-PCNSs-5 were determined as 583.8，542.1 and 614.8 $m^2\ g^{-1}$, respectively. The pore sizes of PCNSs, N-PCNSs-3 and N-PCNSs-5 were determined as 2.2, 3.0 and 3.1 nm, respectively. These data indicated that addition of melamine did not cause obvious difference in surface area and porous structure of PCNSs.
